# Supplementary material for: Lack of riluzole efficacy in the progression of the neurodegenerative phenotype in a new conditional mouse model of striatal degeneration
Source: PeerJ. 2017 Apr 27;5:e3240. doi: 10.7717/peerj.3240 (PMC5410142; doi:10.7717/peerj.3240)
Supplement: Supplemental Information 1 [file peerj-05-3240-s001.pdf]

mouse	genotype	treatment	1A	1B	avg dupl	avg n	SEM
2953	con	veh	40	74	57	**65**	10.0
2961	con	veh	60	7	34		
2977	con	veh	96	34	65		
2990	con	veh	57	114	86		
2955	con	veh	50	48	49		
2956	con	veh	72	130	101		
2959	con	RIL	93	85	89	**109**	15.4
2979	con	RIL	66	87	77		
2980	con	RIL	65	108	87		
2993	con	RIL	164	137	151		
2995	con	RIL	155	128	142		
2947	mut	veh	82	106	94	**118**	18.9
2976	mut	veh	70	106	88		
2992	mut	veh	171	171	171		
2988	mut	veh	127	108	118		
2944	mut	RIL	194	151	173	**133**	19.1
2942	mut	RIL	73	102	88		
2964	mut	RIL	70	89	80		
2971	mut	RIL	178	178	178		
2972	mut	RIL	178	117	148		
