# Supplementary material for: Lack of riluzole efficacy in the progression of the neurodegenerative phenotype in a new conditional mouse model of striatal degeneration
Source: PeerJ. 2017 Apr 27;5:e3240. doi: 10.7717/peerj.3240 (PMC5410142; doi:10.7717/peerj.3240)
Supplement: Supplemental Information 2 [file peerj-05-3240-s002.pdf]

mouse	sex	Cre	TIFflox	ID	treat	time [s]	avg	SEM
2940	F	cre-	+/d	021	VEH	138	**218.3**	22.9
2960	F	cre+	+/+	023	VEH	193		
2977	F	cre+	+/+	025	VEH	290		
2987	F	cre-	d/d	028	VEH	290		
2990	F	cre-	+/d	028	VEH	179		
2953	M	cre+	+/+	023	VEH	229		
2955	M	cre+	+/+	023	VEH	142		
2956	M	cre-	+/d	023	VEH	285		
2941	F	cre-	+/d	021	**RIL**	235	**194.9**	15.9
2944	F	cre-	+/d	021	**RIL**	244		
2979	F	cre-	+/d	026	**RIL**	169		
2980	F	cre-	+/d	026	**RIL**	220		
2958	M	cre-	+/+	023	**RIL**	123		
2959	M	cre-	+/+	023	**RIL**	189		
2995	M	cre-	+/d	029	**RIL**	184		
**2947**	**F**	**cre+**	**d/d**	**022**	VEH	186	**129.1**	20.6
**2948**	**F**	**cre+**	**d/d**	**022**	VEH	78		
**2961**	**F**	**cre+**	**d/d**	**023**	VEH	76		
**2976**	**F**	**cre+**	**d/d**	**025**	VEH	130		
**2988**	**F**	**cre+**	**d/d**	**028**	VEH	215		
**2992**	**F**	**cre+**	**d/d**	**028**	VEH	87		
**2946**	**M**	**cre+**	**d/d**	**022**	VEH	132		
**2942**	**F**	**cre+**	**d/d**	**021**	**RIL**	43	**114.0**	19.2
**2949**	**F**	**cre+**	**d/d**	**022**	**RIL**	136		
**2950**	**F**	**cre+**	**d/d**	**022**	**RIL**	111		
**2963**	**M**	**cre+**	**d/d**	**024**	**RIL**	105		
**2964**	**M**	**cre+**	**d/d**	**024**	**RIL**	195		
**2971**	**M**	**cre+**	**d/d**	**025**	**RIL**	65		
**2972**	**M**	**cre+**	**d/d**	**025**	**RIL**	143		

n
8
7
7
7
