# Supplementary material for: Lack of riluzole efficacy in the progression of the neurodegenerative phenotype in a new conditional mouse model of striatal degeneration
Source: PeerJ. 2017 Apr 27;5:e3240. doi: 10.7717/peerj.3240 (PMC5410142; doi:10.7717/peerj.3240)
Supplement: Supplemental Information 3 [file peerj-05-3240-s003.pdf]

Genotyp	Treatment	Sex	spines/um
con	RIL	F	0.85812008
con	RIL	F	1.01373458
con	RIL	F	0.94840561
con	RIL	M	0.81910466
con	VEH	F	0.95143664
con	VEH	F	1.04147029
con	VEH	M	1.00281865
con	VEH	M	0.87163497
con	VEH	M	0.84009656
**mut**	RIL	**F**	0.93284992
**mut**	RIL	**M**	1.00661395
**mut**	RIL	**M**	0.86628784
**mut**	VEH	**F**	1.0899245
**mut**	VEH	**F**	0.96809213
**mut**	VEH	**M**	0.88800899
**mut**	VEH	**M**	0.82893192
